# Association between extreme temperature and acute myocardial infarction hospital admissions in Beijing, China: 2013–2016

**DOI:** 10.1371/journal.pone.0204706

**Published:** 2018-10-17

**Authors:** Xiaole Liu, Dehui Kong, Jia Fu, Yongqiao Zhang, Yanbo Liu, Yakun Zhao, Hui Lian, Xiaoyi Zhao, Jun Yang, Zhongjie Fan

**Affiliations:** 1 Department of Cardiology, Peking Union Medical College Hospital, Peking Union Medical College, Chinese Academy of Medical Sciences, Beijing, China; 2 Department of Physical Medicine and Rehabilitation, Peking Union Medical College Hospital, Peking Union Medical College, Chinese Academy of Medical Sciences, Beijing, China; 3 Institute for Environmental and Climate Research, Jinan University, Guangzhou, China; Edith Cowan University, AUSTRALIA

## Abstract

Over the past few decades, a growing body of epidemiological studies found the effects of temperature on cardiovascular disease, including the risk for acute myocardial infarction (AMI). Our study aimed to investigate whether there is an association between extremely temperature and acute myocardial infarction hospital admission in Beijng, China. We obtained 81029 AMI cases and daily temperature data from January 1, 2013 to December 31, 2016. We employed a time series design and modeled distributed lag nonlinear model (DLNM) to analyze effects of temperature on daily AMI cases. Compared with the 10th percentile temperature measured by daily mean temperature (Tmean), daily minimum temperature (Tmin) and daily minimum apparent temperature (ATmin), the cumulative relative risks (CRR) at 1st percentile of Tmean, Tmin and ATmin for AMI hospitalization were 1.15(95% CI: 1.02, 1.30), 1.24(95% CI: 1.11, 1.38) and 1.41(95% CI: 1.18, 1.68), respectively. Moderate low temperature (10th vs 25th) also had adverse impact on AMI events. The susceptive groups were males and people 65 years and older. No associations were found between high temperature and AMI risk. The main limitation of the study is temperature exposure was not individualized. These findings on cold-associated AMI hospitalization helps characterize the public health burden of cold and target interventions to reduce temperature induced AMI occurrence.

## Introduction

From 1956 to 2005, the global average temperature rose by 0.13 °C per decade, and the number was 0.07 °C between 1906 to 1956[1]. According to a World Health Organization survey, besides the increased temperature, the frequency and intensity of extreme weather have also changed. The Intergovernmental Panel on Climate Change has demonstrated the effects of temperature variations (extreme heat episodes, sudden cold) on human health, including the effects on temperature- sensitive diseases.

Previous studies showed that both high and low temperature can significantly increase hospital admissions[2–6] and mortality for acute myocardial infarction[7–10]. The underlying mechanism was hypothesized to be increased sympathetic nervous activity, higher blood pressure, heart rate, left ventricular end-diastolic pressure and myocardial oxygen consumption, as well as reduced ischemia threshold, the change of hemodynamics and coagulation[10–12]. A review and meta-analysis of the association between ambient temperature and the incidence of myocardial infarction indicated that both hot and cold weather had detrimental effects on the short-term risk of myocardial infarction[13]. A study conducted in the United States between 1989 and 2000 showed, in extremely cold and hot weather coronary mortality increased 1.59% (95%CI: 0.56, 2.63) and 5.74% respectively (95%CI: 3.38, 8.15). Moreover, the effect of extremely cold weather on acute coronary syndrome is more obvious[10].

Previous studies on temperature and AMI have been conducted mostly in the developed countries rather than the developing ones. China is a highly populated country, with a broad terrain, changeful climate, and at the same time, high incidence of cardiovascular diseases (CVD). According to the report on CVD epidemiology in China, as many as 3.5 million people die from cardiovascular diseases each year, accounting for 41% of all deaths[14]. Beijing is the capital of China, with a population around 20 million, where a complete disease reporting system has been established. Therefore, it is convenient to study temperature-related health effects here.

This study examined the association between extreme temperature and AMI hospital admission in China for the first time. Indicators of extreme temperature included daily maximum temperature (Tmax), the apparent temperature of the daily maximum temperature (ATmax), daily minimum temperature (Tmin), the apparent temperature of the daily minimum temperature (ATmin), as well as low temperature and high temperature range of daily average temperature (Tmean). We also evaluated how demographic factors such as gender and age influenced the effects of temperature. The goal of the study, on a broader prospect, is to illustrate extreme weather’s impact on cardiovascular diseases and potentially better its outcomes by early forecast and intervention.

## Method

### Study population and outcomes

The data of AMI hospital admission from January 1, 2013 to December 31, 2016 was collected by the public health information center of Beijing. The center collected hospitalization data from major hospitals in Beijing and categorize it by diagnosis according to the 10^th^ edition of International Classification of Diseases (ICD-10). The studied category was AMI (ICD-10: I21-22). Each AMI record includes hospital name, patient’s gender, age, birthplace, current address, registration address, work address, admission date, the main discharge diagnosis etc. The study was approved by the ethical committee from Peking Union Medical College Hospital. All data were fully anonymized before we accessed them.

### Meteorological data and air pollution data

Meteorological data was obtained by Chinese meteorological bureau, including Tmean, Tmin, Tmax, relative humidity (RH), wind speed (WS) and air pressure (AP). The apparent temperature (AT), firstly proposed by Steadman, is an indicator for the perception of outdoor temperature[15]. It doesn’t only reflect the outside temperature, but also combine relative humidity and wind speed. Kunst et al. suggested that Steadman's AT is a better measure of human response to wind-chill related stress in cold season than simple ambient air temperature or other thermal indices[16]. Also in hot weather, when humidity is high and wind speed is low, sweat cannot evaporate and the body cools itself at a slower rate, so the actual perceived temperature is higher. We also introduced ATmin and ATmax to our study as measurements of temperature. The following formula was used for conversion.

ATmin/max=Tmin/max+0.33×e-0.70×WS-4.00 (1)

In the above formula, e refers to water vapor pressure and is calculated by the following formula.

e=RH÷100×6.105×exp(17.27×Tmin/max÷(237.7+Tmin/max)) (2)

To allow adjustment for the effects of air pollutants on AMI events, air quality index (AQI), which can reflect the air quality, as well as the influence of all pollutants, was obtained from the air quality monitoring center.

### Statistical analysis

Previous studies suggested that the relationship between temperature and AMI incidences had a lag-effect and was non-linear (U-shaped, V-shaped or J-shaped curve)[17,18]. The distributed lag nonlinear model (DLNM), which was first be used by Armstrong to evaluate the health effect of temperature in 2006[19], was applied to describe exposure-response relationship and lag-response relationship. We controlled for long-term trends using a natural cubic spline with 7 degrees of freedom (df) for the time [20], RH, WS, AP and AQI using a natural cubic spline with 3 df [8,20]. Day of the week (DOW) was used as an indicator variable controlling for variations among days of the week. To analyze the temperature-AMI hospitalization relationship, we used natural cubic spline for temperature (with 4 df, including three internal knots at the 25th, 50thand 75th percentiles of temperatures) and natural cubic for lags (4 df)[21].

In order to fully capture the overall temperature effects and adjust for a possible harvesting effect, we estimated the cumulative effects of ATmin and ATmax on AMI hospital admission within 21 days by using DLNM, and then constructed their exposure-response curves.

Additionally, we evaluated the RRs for the associations between cold and hot temperature and AMI hospital admission by considering relative temperature changes. Cumulative relative risk (CRR) is the cumulative effect obtained by accumulating the relative risk of each day in the lag days. For relative temperature changes of cold, we quantified the CRRs at extremely low temperatures (1st vs. 10th percentile temperature) and moderately low temperatures(10th vs. 25th percentile temperature). For relative temperature changes of hot, we quantified the CRRs at extremely high temperature (99th vs. 90th percentile temperature) and moderately high temperature (90th vs. 75th percentile temperature). These temperatures were calculated based on Tmean, ATmin and ATmax, separately. Two hypothesis tests (e.g. 1st vs. 10th and 10th vs. 25th) were conducted in each data set (e.g. exposure to low temperature measured by Tmin in total subjects). Therefore, according to Bonferroni correction, the significance level should be adjusted to α = 0.05/2 = 0.025.

We also did subgroup analyses stratified by gender (male and female) and age (≤64 years and ≥65 years) to quantitatively assess the cumulative cold and hot effects of temperature on AMI hospital admissions of different populations.

Statistical analyses were conducted using the R software (V.3.4.3) with the ‘dlnm’ package.

## Results

There was a total of 81,029 AMI cases (median of daily cases: 54) in Beijing over the studied period, including 36,989 cases in the age group below 65 (median of daily cases: 25) and 44,040 cases in the age group 65 and above (median of daily cases: 29). Males had more than twice the total (males 55,669 vs. females 25,360) and the median daily counts (males 37 vs. females 17) than that of females (Table 1).

**Table 1:** Summary statistics of AMI event numbers in Beijing, China (2013–2016).

	N	Daily				
		Min*	P(25)**	P(50)**	P(75)**	Max***
Total	81029	23	46	54	63	152
Gender						
Male	55669	13	31	37	44	104
Female	25360	3	13	17	21	57
Age						
<65	36989	7	21	25	29	70
≥65	44040	9	23	29	36	93

* minimum.

**the 25th, 50th (median) and 75th percentile, respectively.

***maximum.

Descriptive statistics for weather conditions and air pollution are shown in Table 2. The daily mean temperature, daily maximum temperature and daily minimum temperature during our study period were 12.8 °C, 18.9 °C and 7.1 °C, respectively. Other meteorological and air pollution indexes, including relative humidity, barometric pressure and AQI, were 53.4%, 1.16.6 hPa and 123.7, respectively (Table 2).

**Table 2:** Descriptive statistics for weather conditions in Beijing, China (2013–2016).

	Mean±SD	Min*	P(25)**	P(50)**	P(75)**	Max***
Daily mean temperature (°C)	12.8±11.2	-16.0	2.0	14.0	23.0	32.0
Daily maximum temperature(°C)	18.9±11.4	-13	8.0	21.0	29.0	42.0
Daily minimum temperature(°C)	7.1±11.3	-17	-3.0	8.0	18.0	27.0
Humidity (%)	53.4±19.9	8.0	38.0	53.0	69.0	97.0
Barometric Pressure (hPa)	1016.6±10.2	994.0	1008.0	1016.0	1025.0	1044.0
AQI	123.7±75.2	23.0	68.0	104.0	159.0	485.0

* minimum.

**the 25th, 50th (median) and 75th percentile, respectively.

***maximum.

**Fig 1:**
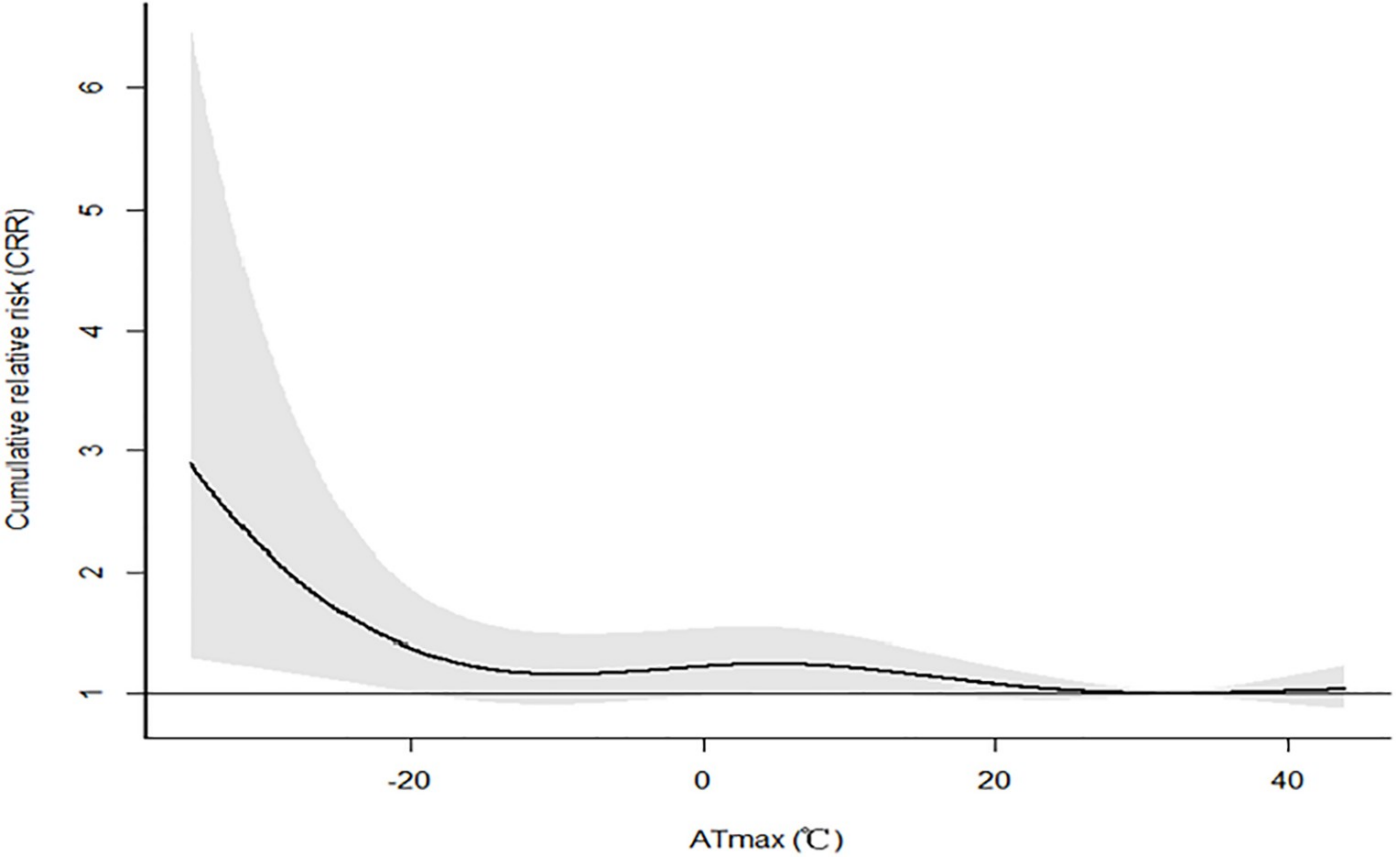
The cumulative effects of ATmax on AMI events over lag 21days in Beijing.

**Fig 2:**
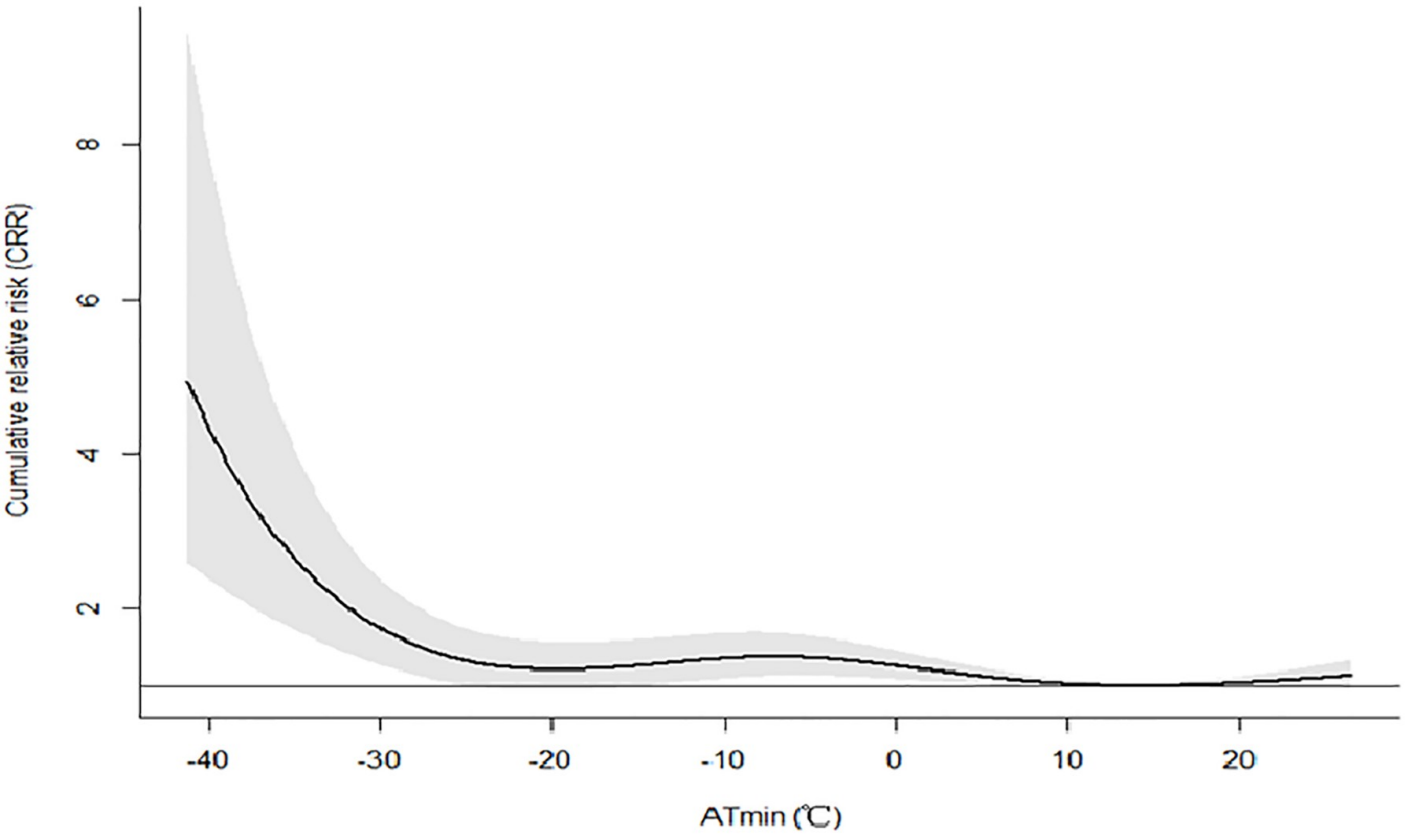
The cumulative effects of ATmin on AMI events over lag 21days in Beijing.

The cumulative effects of ATmax and ATminon AMI events over lag 21dayswere showed in Figs 1 and 2. The CRR was high in extremely low temperature, and had a declining trend as temperature rose. For both ATmax and ATmin, when the temperature was low enough, the risk of AMI hospitalization was noted to be increased. However, high temperature had no effect on AMI occurrence.

Table 3 shows the cumulative effects of low temperature on AMI hospitalization across a 21-day lag period in Beijing. Compared with days with the moderate low temperature (10th *vs* 25th), cold days with extreme low temperature (1st *vs* 10th) had greater effects on AMI hospitalization. The increased cumulative effects of extreme temperature at ATmin, Tmin and Tmean were 1.41(95% CI: 1.18, 1.68), 1.24(95% CI: 1.11, 1.38) and 1.15(95% CI: 1.02, 1.30) respectively, while these of the moderate temperate were 0.94(95% CI: 0.88, 0.99), 0.98(95% CI: 0.92, 1.05) and 0.99(95% CI: 0.94, 1.04) respectively. In addition, the effects of low temperature of ATmin and Tmin on AMI occurrences were greater compared to low temperature of Tmean. The changed trends of CRR from low temperature in subgroups were consistent with the total study population. However, males and patients over 65 years old were more sensitive to the adverse AMI effects of low temperature, compared with females and patients younger than 65 years old.

**Table 3:** The cumulative effects of low temperature on AMI events over lag 21days in Beijing.

	Tmin				ATmin				Tmean			
	1^st^vs 10 ^th^	*p*	10^th^ vs 25^th^	*P*	1^st^ vs 10 ^th^	*p*	10^th^ vs 25^th^	*p*	1^st^ vs 10 ^th^	*P*	10^th^vs 25^th^	*p*
Total	1.24(1.11,1.38)	0	0.98(0.92,1.05)	1.49	1.41(1.18,1.68)	0	0.94(0.88,0.99)	1.98	1.15(1.02,1.30)	0.02	0.99(0.94,1.04)	1.26
Male	1.24(1.09,1.41)	0	0.99(0.91,1.06)	1.20	1.42(1.16,1.74)	0	0.93(0.87, 1)	1.98	1.18(1.03,1.35)	0.02	0.99(0.94,1.05)	0.51
Female	1.23(1.04,1.47)	0.02	0.97(0.88,1.08)	1.45	1.37(1.05,1.79)	0.03	0.95(0.87,1.04)	1.68	1.09(0.91,1.31)	0.32	0.99(0.91,1.07)	0
<65 years	1.17(1.01,1.35)	0.02	0.98(0.9,1.06)	1.38	1.23(0.98,1.55)	0.08	0.95(0.88,1.02)	1.87	1.11(0.95,1.30)	0.17	1.00(0.94,1.07)	1
≥65 years	1.29(1.12,1.49)	0	0.98(0.9,1.07)	1.38	1.56(1.25,1.95)	0	0.93(0.86, 1)	1.92	1.18(1.02,1.37)	0.03	0.99(0.92,1.05)	1.26

There was no significant association between high temperature and AMI hospitalization (Table 4).

**Table 4:** The cumulative effects of high temperature on AMI events over lag 21days in Beijing.

	Tmax				ATmax				Tmean			
	99^th^ vs 90^th^	*p*	90^th^ vs75^th^	*p*	99^th^ vs 90^th^	*p*	90^th^ vs75^th^	*p*	99^th^ vs 90^th^	*p*	90^th^ vs75^th^	*p*
Total	0.95(0.88,1.03)	1.79	0.96(0.90,1.03)	1.68	1.01(0.93,1.10)	0.80	0.99(0.93,1.06)	1.20	0.98(0.92,1.05)	1.38	0.98(0.90,1.06)	1.38
Male	0.96(0.88,1.05)	1.68	0.96(0.89,1.04)	1.68	1.02(0.93,1.13)	0.69	1.00(0.92,1.08)	1.00	0.98(0.90,1.06)	1.38	0.97(0.88,1.06)	1.45
Female	0.94(0.83,1.05)	1.18	0.95(0.85,1.06)	1.68	1.00(0.87,1.14)	1.00	0.98(0.88,1.09)	1.31	0.99(0.89,1.10)	1.16	0.99(0.87,1.13)	1.13
<65 years	0.97(0.88,1.06)	1.45	0.98(0.9,1.07)	1.38	1.03(0.93,1.15)	0.55	1.01(0.93,1.10)	0.80	0.99(0.91,1.08)	1.20	0.99(0.89,1.09)	1.16
≥65 years	0.94(0.85,1.04)	1.77	0.94(0.86,1.03)	1.77	1.00(0.90,1.11)	1.00	0.97(0.89,1.06)	1.45	0.97(0.89,1.07)	1.45	0.97(0.87,1.07)	1.45

**Fig 3:**
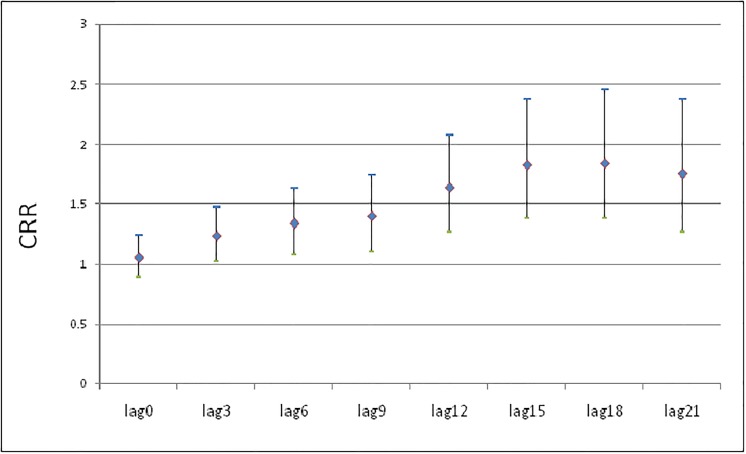
The cumulative effects of 1st of ATmin on AMI hospital admissions.

**Fig 4:**
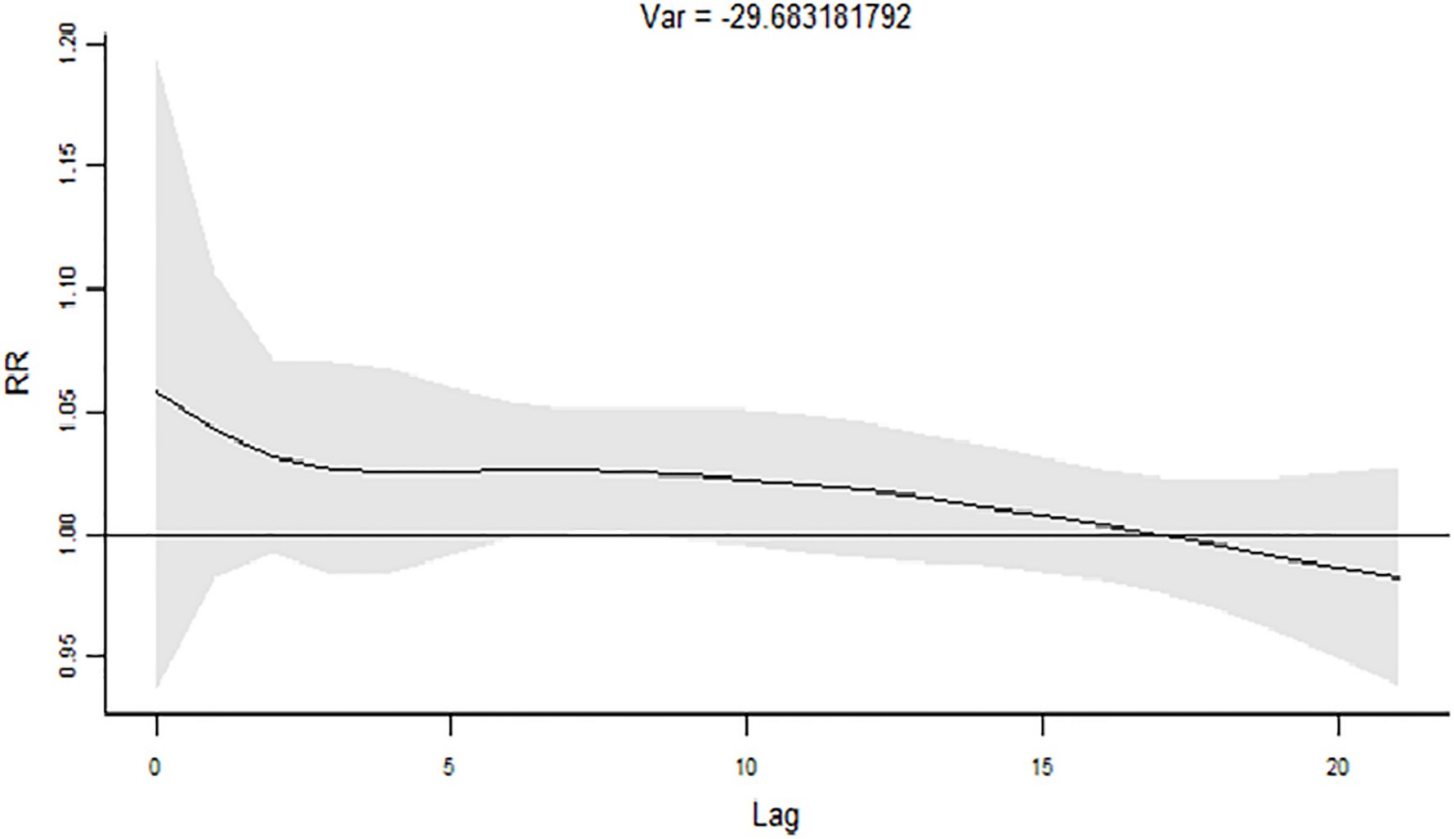
The RR of 1st of ATmin on AMI hospital admissions.

The cumulative effects of 1 st of ATmin on AMI hospital admission are revealed in Fig 3. The CRR of AMI hospitalization gradually increased from lag 0 and reached the maximum value over about lag 17 days. Correspondingly the RR of AMI hospitalization was less than 1 at about lag 17 (Fig 4).

## Discussion

In this large population-based study, statistically significant association was found between short-term exposure to cold temperatures and AMI hospital admissions. Different gender and age groups were shown to have different sensitivity to the cold. High temperature had no effect on the onset of AMI. A total of 81,029 patients with AMI in Beijing provided high statistical power for the analysis and an accurate description of extreme temperature-related effects. The findings have the potential to reduce incidence of AMI and therefore economic burden of government and the family through means of precautions.

Our study found that low temperature can increase the incidence of AMI. This result is consistent with a number of studies conducted in regions with different climate. In a study from 1989 to 2006 in Quebec, Canada, Bayentin L et al. reported that in most of Quebec's regions, cold temperatures during winter months were associated with an increase of up to 12% in the daily hospital admission rate for ischemic heart disease[22]. Panagiotakos DB et al. concluded in their study that there was a significant association between cold weather and increased coronary heart disease incidence[23]. Bhaskaran K et al. suggested in a review that cold weather had detrimental effects on the short-term risk of MI[13]. In a recent study involving 642 patients admitted with AMI, Tsuyoshi Honda and colleagues found that lower minimum temperature on the 2nd day preceding the onset is an independent risk factor for AMI[24]. These findings suggest that cold exposure is a triggering factor for acute cardiac events.

No association was noted between extreme heat and the risk of AMI hospitalization in Beijing. Several previous studies also didn’t found such association[5,6,25,26]. A review included 21 studies showed no apparent association between elevation of temperature and cardiovascular morbidity[27]. Nevertheless, there are different voices on the topic. Bhaskaran K et al. suggested that hot weather had detrimental effects on the short-term risk of MI[13]. Adesuwa S. Ogbomo et al. estimated the extreme-heat-associated hospitalization in Michigan from 2000 to 2009 and observed an increase in hospitalization for AMI at temperatures above the 99th percentile[28]. Jared A. Fisher et al. suggested there is an increased in risk of AMI following extreme heat events in months with warm weather (OR = 1.11; 95% CI: 1.05–1.17) in Maryland[29]. In a case-crossover study from Gothenburg, Sweden, a linear exposure-response corresponding to a 3% and 7% decrease in AMI hospitalization was observed for an inter-quartile range (IQR) increase in the 2-day cumulative average of temperature during the entire year (11°C) and the warm period (6°C), respectively[30]. Although the CRR curve tended to increase in extreme heat in our study, there is no statistical significance. The early warning system of extremely hot weather and the utilization of air conditioning system may have blunted the effect from hot weather.

The effects of extremely low temperature (1st vs10th percentile temperature) measured by Tmean, Tmin and ATmin on AMI hospitalizations were greater than those of moderately low temperature (10th vs25th percentile temperature) of Tmean, Tmin and ATmin in our study. This finding is consistent with the common hypothesis that extremely low temperatures may cause more AMI events since residents there could not adapt to cold. This result suggests that people need to strengthen their adaptation to extreme cold temperature due to local climate changes. In addition, influencing factors of the health effects of extreme cold temperature should be identified so that public health interventions under changing climate could effectively target at-risk groups, mitigate health effects, and improve adaptation.

The effects of extremely low temperature measured by Tmin and ATmin were greater than that of Tmean on AMI incidence. The most likely reason is extremely low temperature of Tmin and ATmin is actually colder and can better reflect daily low temperature than extremely low temperature of Tmean, Tmean is the average temperature of daily Tmin and Tmax. Even though Tmin is very low one day, Tmean can still be high due to the high Tmax and therefore can’t reflect the real low temperature that happened.

We observed that males were more vulnerable to the adverse effects of air pollution on AMI hospitalization at low temperatures. However, previous studies have suggested that low temperature affects females more. One theory proposed is that the cold weather causes the contraction of blood vessels in the skin and enhanced activity of α-epinephrine[31]. Bailey SR et al. found cold-induced activation of Rho/Rho kinase can mediate cold-induced constriction in cutaneous arteries by enabling translocation of alpha2C-ARs to the plasma membrane and by increasing the calcium sensitivity of the contractile process in mice[32]. An estrogen-dependent increase in expression of cold-sensitive alpha(2C)-ARs may contribute to the increased activity of cold-induced vasoconstriction under estrogen-replete conditions[33]. At the same time, estrogen may enhance the expression of this receptor on the surface of large vessel wall. Therefore, in theory females are more sensitive to cold. This inconsistency between our results and theoretical results may be explained the following two reasons. Firstly, there were fewer women in our study, and most of them were over 45 years old (postmenopausal), whose estrogen levels decline and estrogen/progestin ratio abnormal, thus the mechanism above for increasing female risk may not apply. Secondly, the outdoor activity of our study population may be different. For example, men usually retire later than women, so the males in our study may spend more time outdoors on the way to and from work in the morning and in the evening, when the temperature is relatively low throughout the day.

The risk of AMI increased with increasing age. As our data showed, people 65 and older were more vulnerable to the effect from cold weather. This is consistent with some studies conducted in the developed countries. On one hand, as the age increases, the body's perception of the outside environment decreases and its self-regulatory ability decrease, yet the elderly can’t sense the change of temperature in time and take corresponding measures like putting on more clothes. On the other hand, central arterial stiffness is higher in the elderly, which may lead to greater increases in myocardial oxygen demand during cold exposure[34]. Furthermore high prevalence of co-morbidities in the old may also contribute to their vulnerability to the cold due to a disturbance of their internal environment.

A large number of studies showed that the cold effect lasted longer and had obvious hysteresis effect. Within 21 days of the selected delay time in our study, CRR increased constantly and reached its maximum in about 17 days. Therefore, the prevention of AMI should not only be the day with low temperature, but also the days after low temperature.

Reduction of adverse cardiovascular effects of temperature will require both personal actions and public policy measures. Possible individual measures include clothes addition when the temperature drops, reduction of outdoor activities in cold weather and use of air conditioning or heating. Weather forecast, including daily Tmean, Tmin, ATmin and Tmax, should be easily available in media (television, newspaper and internet). Early warning should be issued timely when the temperature is extremely low with cardiovascular risk. Moreover, a concerted effort should be made to educate healthcare providers and high risk population about the potential health hazards of temperature changes.

To our best knowledge, this study is the first to explore the association between extreme temperature and AMI hospitalizations in China. It has certain strengths. Firstly, we incorporated a variety of extreme temperature measurements, including ATmin, ATmax, Tmin, Tmax, extreme low/high temperature and moderate low/high temperature, and compared the effects of different temperature measurements. Secondly, DLNM was applied to better reflect the nonlinearity of the exposure-response relationship and lagged effects, so that relative reliable conclusions can be drawn.

As all ecological epidemiology studies, this study has a disadvantage of exposure misclassification. Data on individual exposure was not available, leading to the compromised assumption that the ambient temperature, RH, AP and air pollution measured in the city the same across Beijing. Another limitation is that certain individual factors were not considered, such as individual time activity pattern and complex endogenous/exogenous factors, which influenced AMI hospitalizations. Lastly, the data was only collected in Beijing with a mid-humid continental climate, and therefore the results of this study can only be generalized to countries with the same environmental and socioeconomic characteristics.

## Conclusions

Low temperatures, especially extreme low temperatures, were responsible for the increased risk of AMI hospitalizations and their lag effects could last more than two weeks. Males and people above 65 years old were identified as susceptible subpopulations. Compared with the ambient low temperature, the temperature that people actually feel were usually lower and had greater effects on AMI incidence. No association was found between high temperature and AMI hospitalizations in the study. Collectively, our study provides quantitative evidence of how cold affects health and calls for developing public policies to reduce temperature induced AMI occurrence.

## Supporting information

S1 File Patients data. (XLSX)

S2 File Meteorological and air pollutant data. (XLSX)

## References

[1] Climate Change 2007: The Physical Science Basis. Contribution of Working Group I to the Fourth Assessment Report of the Intergovernmental Panel on Climate Change. Intergovernmental Panel On Climate Change, Cambridge, UK: Cambridge University Press, 2007.

[2] Goggins WB, Chan EY, Yang CY. Weather, pollution, and acute myocardial infarction in Hong Kong and Taiwan. Int J Cardiol 2013;168:243–249. 10.1016/j.ijcard.2012.09.087 23041014

[3] Wichmann J, Ketzel M, Ellermann T, Loft S. Apparent temperature and acute myocardial infarction hospital admissions in Copenhagen, Denmark: A case-crossover study. Environ Health 2012;11:19 10.1186/1476-069X-11-19 22463704 PMC3353865

[4] Bhaskaran K, Armstrong B, Hajat S, Haines A, Wilkinson P, Smeeth L. Heat and risk of myocardial infarction: Hourly level case-crossover analysis of MINAP database. BMJ 2012;345:e8050 10.1136/bmj.e8050 23243290 PMC3521646

[5] Bhaskaran K, Hajat S, Haines A, Herrett E, Wilkinson P, Smeeth L. Short term effects of temperature on risk of myocardial infarction in England and Wales: Time series regression analysis of the Myocardial Ischaemia National Audit Project (MINAP) registry. BMJ 2010;341:c3823 10.1136/bmj.c3823 20699305 PMC2919679

[6] Chang CL, Shipley M, Marmot M, Poulter N. Lower ambient temperature was associated with an increased risk of hospitalization for stroke and acute myocardial infarction in young women. J Clin Epidemiol 2004;57:749–757. 10.1016/j.jclinepi.2003.10.016 15358404

[7] Hopstock LA, Fors AS, Bonaa KH, Mannsverk J, Njolstad I, Wilsgaard T. The effect of daily weather conditions on myocardial infarction incidence in a subarctic population: The Tromso Study 1974–2004. J Epidemiol Community Health 2012;66:815–820. 10.1136/jech.2010.131458 21652517

[8] Gasparrini A, Armstrong B, Kovats S, Wilkinson P. The effect of high temperatures on cause-specific mortality in England and Wales. Occup Environ Med 2012;69:56–61. 10.1136/oem.2010.059782 21389012

[9] Dilaveris P, Synetos A, Giannopoulos G, Gialafos E, Pantazis A, Stefanadis C. CLimate Impacts on Myocardial infarction deaths in the Athens TErritory: The CLIMATE study. Heart 2006;92:1747–1751. 10.1136/hrt.2006.091884 16840509 PMC1861268

[10] Danet S, Richard F, Montaye M, Beauchant S, Lemaire B, Graux C, et al Unhealthy effects of atmospheric temperature and pressure on the occurrence of myocardial infarction and coronary deaths. A 10-year survey: The Lille-World Health Organization MONICA project (Monitoring trends and determinants in cardiovascular disease). Circulation 1999;100:E1–E7. 10393689 10.1161/01.cir.100.1.e1

[11] Spencer FA, Goldberg RJ, Becker RC, Gore JM. Seasonal distribution of acute myocardial infarction in the second National Registry of Myocardial Infarction. J Am Coll Cardiol 1998;31:1226–1233. 9581712 10.1016/s0735-1097(98)00098-9

[12] Marchant B, Ranjadayalan K, Stevenson R, Wilkinson P, Timmis AD. Circadian and seasonal factors in the pathogenesis of acute myocardial infarction: The influence of environmental temperature. Br Heart J 1993;69:385–387. 8518058 10.1136/hrt.69.5.385 PMC1025097

[13] Bhaskaran K, Hajat S, Haines A, Herrett E, Wilkinson P, Smeeth L. Effects of ambient temperature on the incidence of myocardial infarction. Heart 2009;95:1760–1769. 10.1136/hrt.2009.175000 19635724

[14] Weiwei C, Runlin G, Lisheng L, Manlu Z, Wen W, Yongjun W, et al Outline of the report on cardiovascular diseases in China, 2014. Eur Heart J Suppl 2016;18:F2–F11. 10.1093/eurheartj/suw030 28533724

[15] Krstic G. Apparent temperature and air pollution vs. Elderly population mortality in Metro Vancouver. Plos One 2011;6:e25101 10.1371/journal.pone.0025101 21980381 PMC3182192

[16] Kunst AE, Groenhof F, Mackenbach JP. The association between two windchill indices and daily mortality variation in the Netherlands. Am J Public Health 1994;84:1738–1742. 7977910 10.2105/ajph.84.11.1738 PMC1615202

[17] Chen R, Wang C, Meng X, Chen H, Thach TQ, Wong CM, et al Both low and high temperature may increase the risk of stroke mortality. Neurology 2013;81:1064–1070. 10.1212/WNL.0b013e3182a4a43c 23946311 PMC3795588

[18] Wang XY, Barnett AG, Hu W, Tong S. Temperature variation and emergency hospital admissions for stroke in Brisbane, Australia, 1996–2005. Int J Biometeorol 2009;53:535–541. 10.1007/s00484-009-0241-4 19506912

[19] Armstrong B. Models for the relationship between ambient temperature and daily mortality. Epidemiology 2006;17:624–631. 10.1097/01.ede.0000239732.50999.8f 17028505

[20] Gasparrini A, Armstrong B, Kenward MG. Distributed lag non-linear models. Stat Med 2010;29:2224–2234. 10.1002/sim.3940 20812303 PMC2998707

[21] Gasparrini A, Guo Y, Hashizume M, Lavigne E, Zanobetti A, Schwartz J, et al Mortality risk attributable to high and low ambient temperature: A multicountry observational study. Lancet 2015;386:369–375. 10.1016/S0140-6736(14)62114-0 26003380 PMC4521077

[22] Bayentin L, El AS, Ouarda TB, Gosselin P, Doyon B, Chebana F. Spatial variability of climate effects on ischemic heart disease hospitalization rates for the period 1989–2006 in Quebec, Canada. Int J Health Geogr 2010;9:5 10.1186/1476-072X-9-5 20144187 PMC2830188

[23] Panagiotakos DB, Chrysohoou C, Pitsavos C, Nastos P, Anadiotis A, Tentolouris C, et al Climatological variations in daily hospital admissions for acute coronary syndromes. Int J Cardiol 2004;94:229–233. 10.1016/j.ijcard.2003.04.050 15093986

[24] Honda T, Fujimoto K, Miyao Y. Influence of weather conditions on the frequent onset of acute myocardial infarction. J Cardiol 2016;67:42–50. 10.1016/j.jjcc.2015.02.013 25868809

[25] Bai L, Li Q, Wang J, Lavigne E, Gasparrini A, Copes R, et al Increased coronary heart disease and stroke hospitalisations from ambient temperatures in Ontario. Heart 2018;104:673–679. 10.1136/heartjnl-2017-311821 29101264 PMC5890650

[26] Wolf K, Schneider A, Breitner S, von Klot S, Meisinger C, Cyrys J, et al Air temperature and the occurrence of myocardial infarction in Augsburg, Germany. Circulation 2009;120:735–742. 10.1161/CIRCULATIONAHA.108.815860 19687361

[27] Turner LR, Barnett AG, Connell D, Tong S. Ambient temperature and cardiorespiratory morbidity: A systematic review and meta-analysis. Epidemiology 2012;23:594–606. 10.1097/EDE.0b013e3182572795 22531668

[28] Ogbomo AS, Gronlund CJ, O'Neill MS, Konen T, Cameron L, Wahl R. Vulnerability to extreme-heat-associated hospitalization in three counties in Michigan, USA, 2000–2009. Int J Biometeorol 2017;61:833–843. 10.1007/s00484-016-1261-5 27796569 PMC5410403

[29] Fisher JA, Jiang C, Soneja SI, Mitchell C, Puett RC, Sapkota A. Summertime extreme heat events and increased risk of acute myocardial infarction hospitalizations. J Expo Sci Environ Epidemiol 2017;27:276–280. 10.1038/jes.2016.83 28176761

[30] Wichmann J, Rosengren A, Sjoberg K, Barregard L, Sallsten G. Association between ambient temperature and acute myocardial infarction hospitalisations in Gothenburg, Sweden: 1985–2010. Plos One 2013;8:e62059 10.1371/journal.pone.0062059 23646115 PMC3639986

[31] Flavahan NA, Lindblad LE, Verbeuren TJ, Shepherd JT, Vanhoutte PM. Cooling and alpha 1- and alpha 2-adrenergic responses in cutaneous veins: Role of receptor reserve. Am J Physiol 1985;249:H950–H955. 10.1152/ajpheart.1985.249.5.H950 2865900

[32] Bailey SR, Eid AH, Mitra S, Flavahan S, Flavahan NA. Rho kinase mediates cold-induced constriction of cutaneous arteries: Role of alpha2C-adrenoceptor translocation. Circ Res 2004;94:1367–1374. 10.1161/01.RES.0000128407.45014.58 15087420

[33] Eid AH, Maiti K, Mitra S, Chotani MA, Flavahan S, Bailey SR, et al Estrogen increases smooth muscle expression of alpha2C-adrenoceptors and cold-induced constriction of cutaneous arteries. Am J Physiol Heart Circ Physiol 2007;293:H1955–H1961. 10.1152/ajpheart.00306.2007 17644575

[34] Hess KL, Wilson TE, Sauder CL, Gao Z, Ray CA, Monahan KD. Aging affects the cardiovascular responses to cold stress in humans. J Appl Physiol (1985) 2009;107:1076–1082. 19679742 10.1152/japplphysiol.00605.2009 PMC2763834

